# Reduced cAMP, Akt Activation and p65-c-Rel Dimerization: Mechanisms Involved in the Protective Effects of mGluR3 Agonists in Cultured Astrocytes

**DOI:** 10.1371/journal.pone.0022235

**Published:** 2011-07-14

**Authors:** Daniela Durand, Lila Carniglia, Carla Caruso, Mercedes Lasaga

**Affiliations:** Research Institute for Reproduction, School of Medicine, University of Buenos Aires, Ciudad Autónoma de Buenos Aires, Beunos Aires, Argentina; University of California Los Angeles, and Cedars-Sinai Medical Center, United States of America

## Abstract

In recent decades, astrocytes have emerged as key pieces in the maintenance of normal functioning of the central nervous system. Any impairment in astroglial function can ultimately lead to generalized disturbance in the brain, thus pharmacological targets associated with prevention of astrocyte death are actually promising. Subtype 3 of metabotropic glutamate receptors (mGluR3) is present in astrocytes, its activation exerting neuroprotective roles. In fact, we have previously demonstrated that mGluR3 selective agonists prevent nitric oxide (NO)-induced astrocyte death. However, mechanisms responsible for that cytoprotective property are still subject to study. Although inhibition of adenylyl cyclase by mGluR3 activation was extensively reported, the involvement of reduced cAMP levels in the effects of mGluR3 agonists and the association between cAMP decrease and the downstream pathways activated by mGluR3 remain neglected. Thus, we studied intracellular signaling mediating anti-apoptotic actions of mGluR3 in cultured rat astrocytes exposed to NO. In the present work, we showed that the cytoprotective effect of mGluR3 agonists (LY379268 and LY404039) requires both the reduction of intracellular cAMP levels and activation of Akt, as assessed by MTT and TUNEL techniques. Moreover, dibutyryl-cAMP impairs Akt phosphorylation induced by LY404039, indicating a relationship between mGluR3-reduced cAMP levels and PI3K/Akt pathway activation. We also demonstrated, by co-immunoprecipitation followed by western-blot, that the mGluR3 agonists not only induce *per se* survival-linked interaction between members of the NF-κB family p65 and c-Rel, but also impede reduction of levels of p65-c-Rel dimers caused by NO, suggesting a possible anti-apoptotic role for p65-c-Rel. All together, these data suggest that mGluR3 agonists may regulate cAMP/Akt/p65-c-Rel pathway, which would contribute to the protective effect of mGluR3 against NO challenge in astrocytes. Our results widen the knowledge about mechanisms of action of mGluR3, potential targets for the treatment of neurodegenerative disorders where a pathophysiological role for NO has been established.

## Introduction

Normal function of the central nervous system (CNS) depends on adequate maintenance of the neuronal microenvironment. This requires, in turn, the presence and correct functioning of astrocytes, which regulate extracellular ionic composition, remove neurotransmitter excess at the synaptic cleft and contribute to functional hyperemia in active brain tissue, among several other essential functions [1]. Therefore, loss of astroglia or impairment in astroglial function can lead to generalized disturbance in the brain. We have demonstrated that the inflammatory stimulus of bacterial lipopolysaccharide (LPS)+interferon-γ (IFN-γ) induces astroglial death, which is mediated by nitric oxide (NO) production [2], [3]. Moreover, an NO donor, DETA/NO, also induces astrocyte death [3].

Metabotropic glutamate receptors (mGluR) belong to the family of G-protein coupled receptors. Eight mGluR subtypes have been cloned and are classified into groups I (mGluR1 and 5), II (mGluR2 and 3) and III (mGluR4, 6, 7 and 8). Groups II and III are negatively coupled to adenylyl cyclase, thereby inhibiting cyclic AMP (cAMP) formation [4]. Several reports show mGluR expression in glial cells. Of group II mGluRs, only the mGluR3 subtype was found in astrocytes [5] where it may have a protective role. In fact, agonists of group II mGluR are more effective against excitotoxic death in mixed neuron-glia cultures than in pure neuronal cultures [6]. Activation of group II mGluR stimulates release of neuroprotective factors such as brain derived neurotrophic factor and transforming growth factor-β (TGF-β) from astrocytes [7]. Moreover, a synthetic mGluR3 agonist, (−) 2-oxa-4-aminocyclo-[3.1.0] hexane-4,6-dicarboxylic acid (LY379268), protects cultured astrocytes against apoptotic death induced by oxygen/glucose deprivation [8]. Concordantly, our previous results demonstrated that mGluR3 activation by LY379268 prevents DETA/NO-induced cell death in primary astrocytes by a mechanism involving p53, Bax and Bcl-2 modulation and prevention of mitochondrial membrane permeabilization [3].

For the present study, we decided to investigate pathways activated by mGluR3 which might mediate the protective actions of these receptors. We evaluated the NF-κB pathway not only because its members are linked to induction of nitric oxide synthase (NOS) transcription but also because it was recently postulated that, depending on the composition of the dimers activated, they may have pro- or anti-apoptotic effects. It is now proposed that p65-p50 heterodimers may lead to apoptosis, whereas c-Rel containing dimers have a protective role [9]. We also studied the PI3K/Akt pathway, which has frequently been associated with mGluR3 activity [10] and has a major role in the induction of survival signals [11], [12]. Finally, we analyzed whether mGluR3-induced inhibition of cAMP production is involved in the anti-apoptotic action of this receptor subtype in astrocytes.

Our results demonstrate that mGluR3-induced cAMP reduction, Akt activation and p65-c-Rel interaction mediate the anti-apoptotic effect of mGluR3 agonists in NO-challenged astrocytes, possibly as part of a unique cAMP/Akt/p65-c-Rel signaling pathway.

## Materials and Methods

### Ethics Statement

All experimental procedures were approved by the Committee on Ethics of the School of Medicine (University of Buenos Aires, Resolution No 1889/06) and were carried out in compliance with the guidelines of the NIH Guide for the Care and Use of Laboratory Animals.

### Materials

The (−)2-oxa-4-aminobicyclo[3.1.0]hexane-4,6-dicarboxylic acid (LY379268) was purchased from TOCRIS Bioscience (MO, USA).

The (−)-(1R,4S,5S,6S)-4-amino-sulfonylbicyclo[3.1.0]-hexane-4,6-dicarboxylic acid (LY404039) was custom-synthesized by Selleck Chemicals (ShangHai, China). Interferon-γ (IFN-γ) was purchased from Boehringer Ingelheim (Buenos Aires, Argentina). Diethylenetriamine nitric oxide adduct (DETA/NO), LPS (*Escherichia coli*, serotype O127:B8), dibutyryl-cAMP, 8-Br-cGMP, Akt inhibitor and anti-β-actin antibody were purchased from Sigma-Aldrich Corporation (MO, USA). Fetal bovine serum (FBS) was obtained from PAA laboratories GmBH (Pasching, Austria). DMEM/F-12, antibiotic and antimycotic were purchased from Invitrogen Life technologies (CA, USA). Anti-c-Rel and anti-histone antibodies and protein A/G agarose were purchased from Santa Cruz Biotechnology (CA, USA). Anti-p65 antibody was purchased from BD Biosciences (CA, USA). Anti-GFAP, anti-mGluR2/3, biotinylated donkey anti-mouse and anti-rabbit antibodies were obtained from Chemicon International Inc. (CA, USA). Anti-phospho-Akt, anti-Akt and anti-IκBα antibodies were obtained from Cell Signalling Technology Inc. (MA, USA). TUNEL reagents were obtained from Roche Diagnostics (Mannheim, Germany).

### Astrocyte primary cultures

Cerebral hemispheres of 1- to 2-day-old postnatal Wistar rat pups were dissected, freed from meninges and cut into small fragments. Tissue was disrupted by triturating it through a needle in DMEM/F-12 medium containing 10% FBS, 50 µg/mL streptomycin, 0.125 µg/mL amphotericin, and 50 U penicillin. Then cells were seeded in 75 cm^2^ poly-L-lysine coated culture flasks. Cultured cells were kept at 37°C in 5% CO_2_. After reaching confluence, astrocytes were separated from microglia and oligodendrocytes by shaking flasks for 24 h in an orbital shaker at 240 rpm. Cells were tripsinized, subcultured and, after 2–3 days of stabilization, incubated with the drugs in MEM containing 2% FBS, 2 mM L-glutamine, 50 µg/mL streptomycin, 0.125 µg/mL amphotericin and 50 U penicillin.

Cultures were routinely more than 95% pure astrocytes when assessed by glial fibrillary acidic protein (GFAP) immunostaining as previously described [2].

### Treatments

Astrocytes were incubated with 1 µg/mL LPS and 50 ng/mL IFN-γ in the presence or absence of LY379268 for 30 min, or with 1 mM DETA/NO in the presence or absence of 100 µM LY379268 or LY404039 (highly selective group II mGluR agonists) and Akt inhibitor (0.5 µM), PI3K/Akt inhibitor (20 µM), db-cAMP (1 mM) or 8Br-cGMP (1 mM) for 30 min or 48 h. When treated with Akt or PI3K/Akt inhibitors, astrocytes were preincubated with the inhibitor alone for 60 min and then treated with the other drugs. Control cells were grown in MEM containing 2% FBS, 2 mM L-glutamine, 50 µg/mL streptomycin, 0.125 µg/mL amphotericin, and 50 U penicillin.

### Double immunocytochemistry for mGluR2/3 and GFAP

2×10^4^ astrocytes were seeded on poly-L-lysinated coverslips in culture medium for 96 h and fixed with 4% formaldehyde in PBS (140 mM NaCl, 10 mM NaH_2_PO_4_/Na_2_HPO_4_, pH 7.4) for 30 min at 4°C. Cells were permeabilized by microwave irradiation in citrate buffer (10 mM, pH 6.0) and blocked with 10% donkey serum and 0.2 mL/mL avidin blocking solution in PBS for 1 h at room temperature (RT). Primary antibodies (anti-mGluR2/3 1∶100 and anti-GFAP 1∶200) were incubated in buffer containing 1% donkey serum and 0.2 mL/mL biotin blocking solution in PBS overnight at 4°C. Then, cells were incubated with secondary antibodies (biotinilated donkey-anti-rabbit IgG 1∶400 and TRITC-conjugated anti-mouse IgG 1∶150) for 1 h at RT, followed by FITC-conjugated avidin 1∶600 (Vector Laboratories) in 10 mM Hepes pH 7.9, for 20 min at RT. Coverslips were mounted with 4′,6-diamino-2-phenylindole (DAPI, Vectashield, Vector Laboratories Inc., CA, USA) and astrocytes were visualized in a fluorescence microscope (Zeiss Axiophot, Germany). Negative control slides were incubated in dilution buffer instead of primary antibody.

### Metabolic activity assay

Metabolic activity of viable cells was measured by the 3-[4,5-dimethylthiazol-2-yl]-2,5-diphenyltetrazolium bromide (MTT) assay. Briefly, cells (4×10^4^) were washed and incubated for 4 h in 100 µL Krebs buffer plus 50 µg of MTT reagent dissolved in 10 µL phosphate buffered saline (PBS) at 37°C. Formazan crystals obtained from MTT reduction were dissolved in 100 µL 0.04 M HCl in isopropanol and optical density (OD) was measured in a microplate spectrophotometer at 595 nm.

### Microscopic determination of DNA fragmentation by terminal deoxynucleotidyl transferase-mediated dUTP nick end labeling (TUNEL) assay

Cells were fixed with 4% formaldehyde in PBS pH 7.4 for 30 minutes and permeabilized by microwave irradiation. DNA strand breaks were labeled with digoxigenin-deoxy-UTP using terminal deoxynucleotidyl transferase (TdT, 0.18 U/µL) as previously described [13]. The incorporation of nucleotides into the 3′-OH end of damaged DNA was detected with an anti-digoxigenin-fluorescein antibody. Slides were mounted with mounting medium for fluorescence containing DAPI for DNA staining and visualized in a fluorescence microscope (Axiophot, Carl Zeiss, Jena, Germany). Negative control slides were incubated in absence of TdT.

### Total protein extracts

1×10^6^ astrocytes per well were scrapped into PBS+10 mM NaF+1 mM Na_3_VO_4_ and centrifuged at 2000 rpm. Pellets were homogenized in lysis buffer (50 mM Tris-HCl pH 7.4, 1 mM EDTA, 150 mM NaCl, 1% NP-40) containing protease and phosphatase inhibitors (1 mM PMSF, 1 µg/mL aprotinin, 1 µg/mL leupeptin, 1 µg/mL pepstatin A, 10 mM NaF, 1 mM Na_3_VO_4_). Following sonication and centrifugation at 12000 rpm for 30 min, the supernatant was assayed by immunoblot. Protein concentration in samples was determined by the Bradford method (BioRad Laboratories, CA, USA) using bovine serum albumin as standard.

### Nuclear and cytosolic extracts

4–5×10^6^ astrocytes were grown in Petri dishes and treated with corresponding drugs for 30 min. Cells were scrapped into PBS-PMSF (1 mM), centrifuged at 2500 rpm and resuspended in 150 µL lysis buffer A (10 mM Hepes, 10 mM KCl, 2 mM MgCl_2_, 500 µM DTT, 1 mM PMSF, 5 µg/mL aprotinin, 5 µg/mL leupeptin, 5 µg/mL pepstatin A, 500 µM Na_3_VO_4_, 1 mM NaF), followed by incubation with 1% NP-40. Homogenates were centrifuged at 14000 rpm for 15 min and supernatants collected as the cytosolic fraction. Pellets were resuspended in buffer B (20 mM Hepes, 25% glycerol, 420 mM NaCl, 1.5 mM MgCl_2_, 200 µM EDTA, 500 µM DTT, plus protease and phosphatase inhibitors) and shaken on ice for 30 min. After centrifugation at 14000 rpm for 15 min, supernatants (nuclear extracts) were diluted in buffer C (20 mM Hepes, 20% glycerol, 50 mM KCl, 200 µM EDTA, 500 µM DTT, 1 mM PMSF). Protein concentration was determined by the Bradford method. As a control of subcellular fractioning, we ran immunoblots of nuclear and cytosolic extracts using anti-histone antibody, detecting a positive band only in the nuclear fraction.

### Western blots

30–50 µg of proteins were size-fractionated in 8% (or 15% for IκBα) sodium dodecyl sulfate (SDS)-polyacrilamide gel, then electrotransferred to a polyvinylidene difluoride membrane. Blots were blocked for 1–2 h in 5% nonfat dry milk-TBS-0.1% Tween 20 and incubated overnight at 4°C with appropriate primary antibodies in 5% milk-TBS-0.1% Tween 20 (anti-p65 1∶400, anti-c-Rel 1∶5000, anti-IκBα 1∶1000) or in 5% BSA- TBS-0.1% Tween 20 (anti-Akt 1∶2000, anti-phospho-Akt 1∶1000). This was followed by 1 h incubation with the respective biotinylated secondary antibody and 1 h incubation with Streptavidin-peroxidase (Chemicon International Inc., CA, USA). Immunoreactivity was detected by enhanced chemiluminescence (ECL plus, Amersham Biosciences, GE Healthcare). Bands were analyzed using SCION Image software. Results were normalized to the internal control β-actin, except for phospho-Akt, which was expressed as phospho-Akt/Akt ratio. Values were expressed relative to respective controls.

### Co-immunoprecipitation from nuclear extracts

50–70 µg of nuclear proteins were diluted up to 500 µL in lysis buffer (50 mM Tris-HCl, 140 mM NaCl, 0.1% NP-40, 1 mM PMSF, 50 µg/mL aprotinin, 2.3 µg/mL leupeptin, 0.5 µg/mL pepstatin A, 0.5 mM Na_3_VO_4_ and 1 mM NaF). We pre-cleared with protein A/G agarose to reduce non-specific adsorption. After centrifuging at 2500 rpm for 10 min at 4°C, supernatants were incubated with 1 µg of anti-c-Rel antibody overnight at 4°C with continuous shaking. 20 µL of protein A/G agarose were added and antigen-antibody complexes precipitated by centrifugation at 2500 rpm for 10 min. Pellets were washed 3 times with washing buffer (50 mM Tris-HCl pH 7.5, 250 mM NaCl, 0.1% NP-40, with protease and phosphatase inhibitors) and the final pellet was resuspended in 25 µL of sample buffer 1X, then loaded onto a SDS-PAGE to run immunoblots for p65. As a control of specificity of immunoprecipitation, nuclear proteins were precipitated with an irrelevant IgG of the same origin as anti-c-Rel (anti-mGluR2/3). As a control of the presence of p65 in nuclear extracts, non-immunoprecipitated extracts were used in western-blots for p65 (input).

### Statistical analysis

Data were expressed as mean ± SEM and analyzed by one sample Student's *t* test, unpaired Student's *t* test, or one-way analysis of variance (ANOVA) followed by Dunnet or Bonferroni Multiple Comparisons Test, when corresponding. For multiple Student's *t* tests we used the Holm adjustment of the significance level. For other statistical tests than the Holm adjustment, the significance level was p<0,05.

## Results

### Presence of mGluR3 in astrocytes cultures

**Figure 1:**
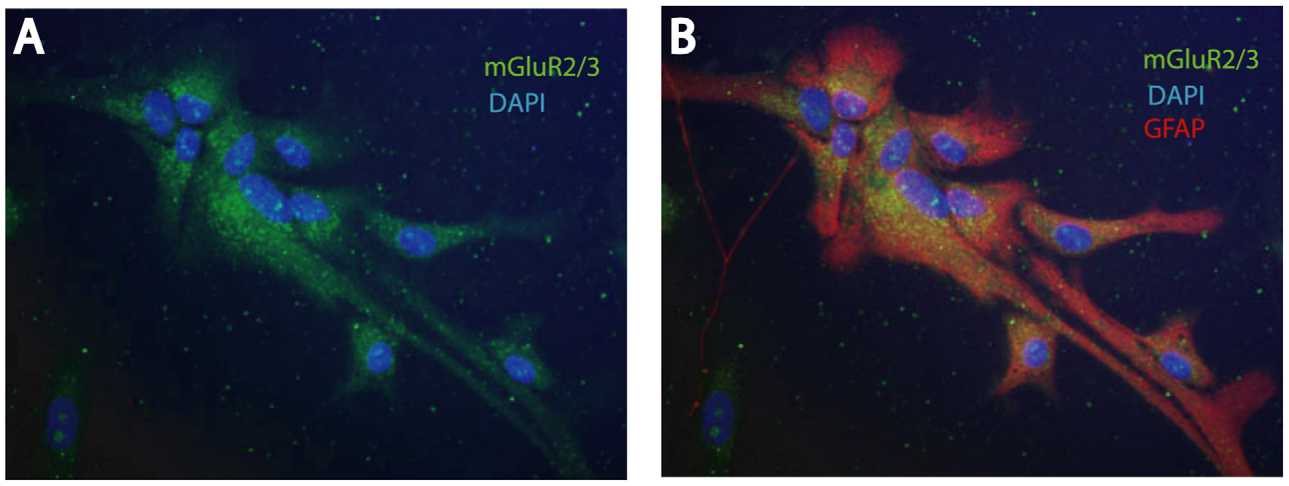
mGluR3 expression in astrocytes. Immunolabeling for mGluR2/3 (green), GFAP (red) and DAPI (blue) done on rat primary astrocytes. (A) Immunolabeling for mGluR2/3. Picture B shows mGluR2/3 and GFAP merged images (yellow).

As previously described [3], [14]–[19] we confirmed the expression of group II mGluRs in rat astrocytes by immunocytochemistry (Fig. 1). Despite the limited availability of selective antibodies for mGluR2 or mGluR3, we postulate that staining obtained with mGluR2/3 antibody corresponds to mGluR3, since only this subtype has been found in astrocytes [20], [21].

### Signaling pathways mediating mGluR3 actions

Once we had demonstrated that astrocytic mGluR3 activation prevents NO-induced astrocyte death and reduces LPS/IFN-γ-stimulated NO synthesis [3], we were interested in elucidating the signaling pathways involved in mGluR3-induced cytoprotection. For this purpose, we used two mGluR3 agonists with similar pharmacological properties: LY379268 and LY404039 [22], [23].

### NF-κB family

Since NF-κB family proteins are intimately associated with iNOS transcription, we analyzed protein expression of several members of this family. As an approach to their transcriptional activity, p65 and c-Rel levels were determined from nuclear extracts, whereas inhibitor IκB levels were determined from cytosolic extracts.

**Figure 2:**
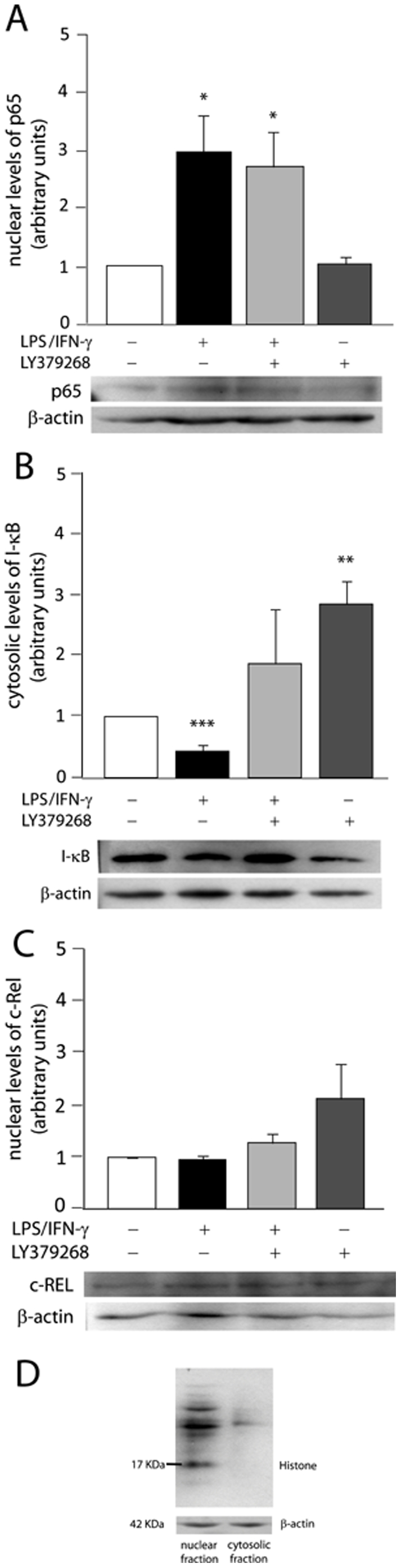
Expression of NF-κB family proteins. Astrocyte primary cultures were exposed to LPS/IFN-γ (1 µg/mL/50 ng/mL)±100 µM LY379268 for 30 min and nuclear and cytosolic proteic extracts were used for western-blots of p65 (A), IκBα (B) and c-Rel (C). Values of OD from bands were normalized to the internal control β-actin and data expressed relative to control group. Bars represent the mean ± SEM of 5–6 (A and B) or 3 (C) independent experiments. *p<0.05, **p<0.01, ***p<0.001 versus control. (D) The purity of nuclear and cytosolic fractions was assayed by western-blot for nuclear histone.

Cultured astrocytes were incubated with LPS/IFN-γ (1 µg/mL/50 ng/mL) in the presence or absence of a selective mGluR3 agonist LY379268 (100 µM) for 30 min. Mentioned concentrations of LPS/IFN-γ and mGluR3 agonist were used all over the experiments. Treatment with LPS/IFN-γ significantly increased nuclear expression of p65, whereas LY379268 had no effect on p65 expression either *per se* or in combination with LPS/IFN-γ (Fig. 2A). However, the agonist did block the inhibitory effect of LPS/IFN-γ on cytosolic IκB levels and simultaneously induced IκB expression *per se* (Fig. 2B). Conversely, neither LPS/IFN-γ nor LY379268 significantly modified c-Rel protein levels (Fig. 2C).

**Figure 3:**
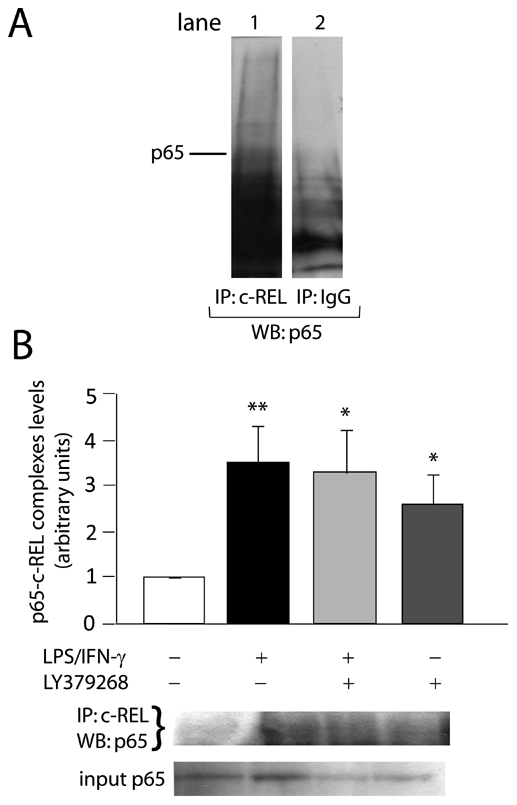
Both LPS/IFN-γ and LY379268 induce p65-c-Rel dimerization. Astrocytes were incubated with LPS/IFN-γ (1 µg/mL/50 ng/mL)±100 µM LY379268 for 30 min. Interaction between p65 and c-Rel proteins was analyzed from 70 µg of proteic nuclear extracts by co-immunoprecipitation (IP) with anti-c-Rel antibody followed by western-blot (WB) for p65. (A) Specificity of the assay was analyzed by immunoprecipitating nuclear proteins with anti-c-Rel antibody (lane 1) or with an irrelevant IgG of the same origin as the anti-c-Rel antibody (lane 2), which was not able to precipitate c-Rel-linked p65. (B) Levels of anti-c-Rel-immunoprecipitated p65 were expressed relative to control group. Bars represent the mean ± SEM of 3 independent experiments. *p<0.05, **p<0.01 versus control. Input corresponds to total content of p65 in nuclear extracts.

A crucial characteristic of NF-κB members is their ability to form homo- and heterodimers. Some authors recently associated p65-p50 dimers with neurodegenerative events and c-Rel-containing dimers with transcription of anti-apoptotic genes [9], [24], [25]. Therefore, we also examined the effect of mGluR3 agonists on p65-c-Rel dimerization by co-immunoprecipitation followed by western-blot. Both LPS/IFN-γ and LY379268 by themselves significantly increased nuclear levels of p65-c-Rel complexes, although they showed no additive effect when combined (Fig. 3B).

**Figure 4:**
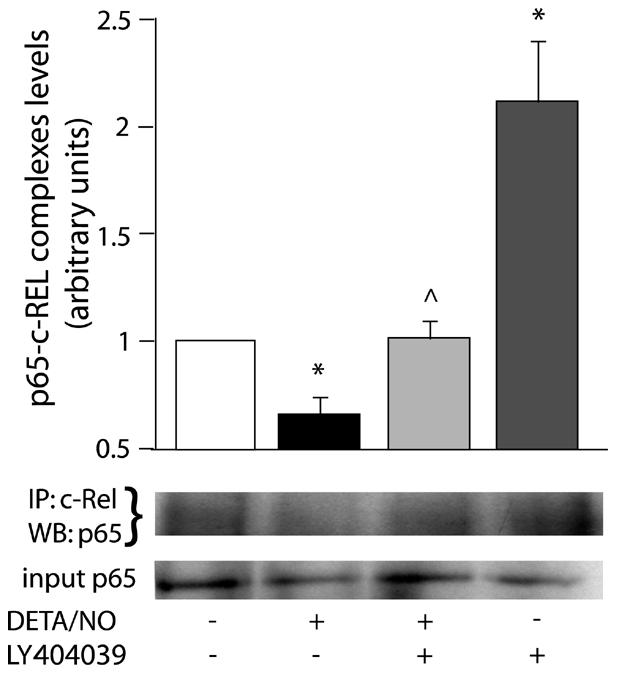
mGluR3 activation avoids inhibition of p65-c-Rel dimerization provoked by DETA/NO. p65-c-Rel interaction was analyzed by co-immunoprecipitation (IP) followed by western-blot (WB) of nuclear extracts from astrocytes treated with 1 mM DETA/NO±100 µM LY404039 for 30 min. Levels of anti-c-Rel-immunoprecipitated p65 were expressed relative to control group. Bars represent the mean ± SEM of 3 independent experiments. *p<0.05 versus control, ∧p<0.05 versus DETA/NO. Input corresponds to total content of p65 in nuclear extracts.

We also determined p65-c-Rel interaction after incubation with pro-apoptotic stimulus DETA/NO (1 mM) for 30 min. It is known that mM amounts of DETA/NO replicate macrophage and reactive astrocyte-generated NO [26], which reachs µM levels. Likewise, our previous data indicate that astrocytes exposed to LPS/IFN-γ release 10-20 µM NO, as measured by Griess Method. Thus, 1 mM DETA/NO is a good approach to mimic NO concentrations released by reactive astrocytes *in vitro* and it was employed throughout the rest of the experiments. In this case, DETA/NO inhibited p65-c-Rel dimerization, whereas another selective mGluR3 agonist, LY404039 (100 µM), restored levels of the complexes (Fig. 4), suggesting a possible protective role of these heterodimers in NO-exposed astrocytes. Like LY379268, LY404039 also induced p65-c-Rel interaction on its own (Fig. 4).

### Involvement of cAMP and cGMP in the effects of mGluR3 activation

**Figure 5:**
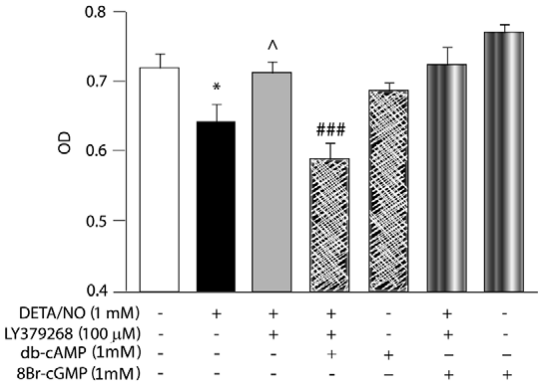
Cytoprotective action of mGluR3 agonists is mediated by reduction of cAMP levels but not by cGMP. Astrocyte primary cultures were incubated with 1 mM DETA/NO in the presence or absence of 100 µM LY379268 and/or 1 mM db-cAMP or 1 mM 8Br-cGMP for 48 h. Cell viability was assayed by the MTT method. Bars represent the mean ± SEM of 6–8 determinations per group, of one representative experiment of 2 independent ones. *p<0.05 versus control; ∧p<0.05 versus DETA/NO; ###p<0.001 versus DETA/NO+LY379268.

Since the most direct action triggered by mGluR3 activation is inhibition of adenylate cyclase and a concomitant reduction in cAMP intracellular levels, we studied the participation of cAMP in protective effects of mGluR3 agonists. Cell viability was measured in astrocytes treated with DETA/NO±LY379268 in the presence or absence of a non-hydrolyzable analog of cAMP, dibutyryl-cAMP (1 mM db-cAMP) for 48 h. As we reported previously [3], DETA/NO significantly reduced astrocyte viability and LY379268 avoided this effect (Fig. 5). Incubation with db-cAMP blocked action of LY379268 on cell viability (Fig. 5), indicating that cAMP has a negative effect on the protection exerted by mGluR3 activity in astrocytes.

As suggested by Wroblewska *et al.* (2006), cGMP (the main second messenger of NO) could be modulated by mGluR3 [27]. We had previously measured cGMP intracellular levels and found that LY379268 did not modify NO-stimulated cGMP levels [3]. Here, we confirmed these results by incubating astrocytes with a non-hydrolyzable analog of cGMP, 8-Br-cGMP (1 mM). This reagent did not alter the protective effect of LY379268 on cell viability reduced by NO (Fig. 5).

Neither db-cAMP nor 8-Br-cGMP affects astrocyte viability *per se* (Fig. 5).

### The Akt pathway

**Figure 6:**
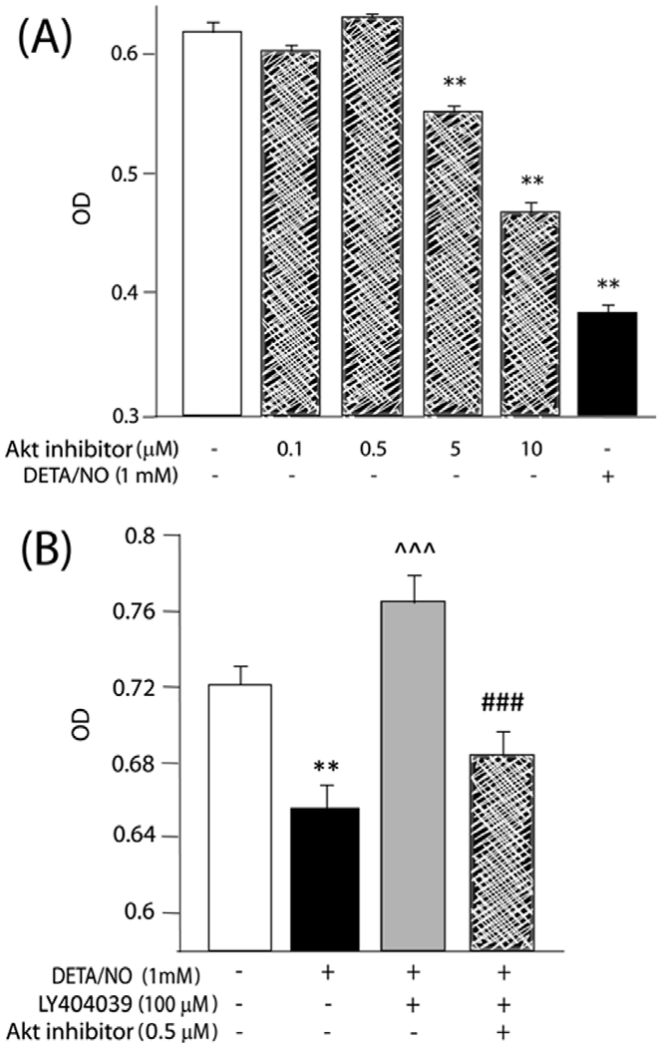
Akt activity mediates the antiapoptotic effect of mGluR3 agonists in astrocytes. (A) A dose-response curve was plotted by a MTT assay to determine the optimal concentration of the Akt inhibitor. (B) Astrocytes were treated with 1 mM DETA/NO in the presence or absence of 100 µM LY404039 and/or Akt inhibitor (0.5 µM) for 48 h. Cell viability was assayed by the MTT method. Bars represent the mean ± SEM of 6–8 determinations per group of one representative experiment of 2 independent ones. **p<0.01 versus control; ∧∧∧p<0.001 versus DETA/NO; ###p<0.001 versus DETA/NO+LY404039.

Several reports now propose the PI3K/Akt system as an intracellular target of mGluR3, even in astrocytes [8], [10]. Therefore, we analyzed involvement of this pathway in our experimental model. Astrocytes were incubated with DETA/NO±LY404039 in the presence or absence of an Akt inhibitor for 48 h and cell viability was assayed. The concentration of the Akt inhibitor used was 0.5 µM, which showed no activity *per se* on cell viability in a dose-response curve (Fig. 6A) and inhibited Akt1 and 2 isoforms, according to previous data [28].

The Akt inhibitor blocked the cytoprotective effect of LY404039 on astrocyte viability reduced by NO (Fig. 6B), indicating that this pathway mediates anti-apoptotic actions of mGluR3 agonists.

The results for db-cAMP and the Akt inhibitor were reproduced by the TUNEL technique, in which both reagents were able to impede the anti-apoptotic effect of the mGluR3 agonist (Table 1).

**Table 1:** Akt activity and cAMP reduction mediate the protective effect of mGluR3 on DNA fragmentation induced by NO.

	CONTROL	DETA/NO	DETA/NO+LY404039	DETA/NO+LY404039+Akt inhibitor	DETA/NO+LY404039+db-cAMP
Percentage of TUNEL positive cells ± SEM	2.44±1.067	6.28±1.207	4.87±0.623	6.01±1.746	6.89±1.395

DNA fragmentation was determined by TUNEL technique, as described in Materials and Methods. Values correspond to the mean ± SEM of the percentage of TUNEL-positive astrocytes of 2 independent experiments.

**Figure 7:**
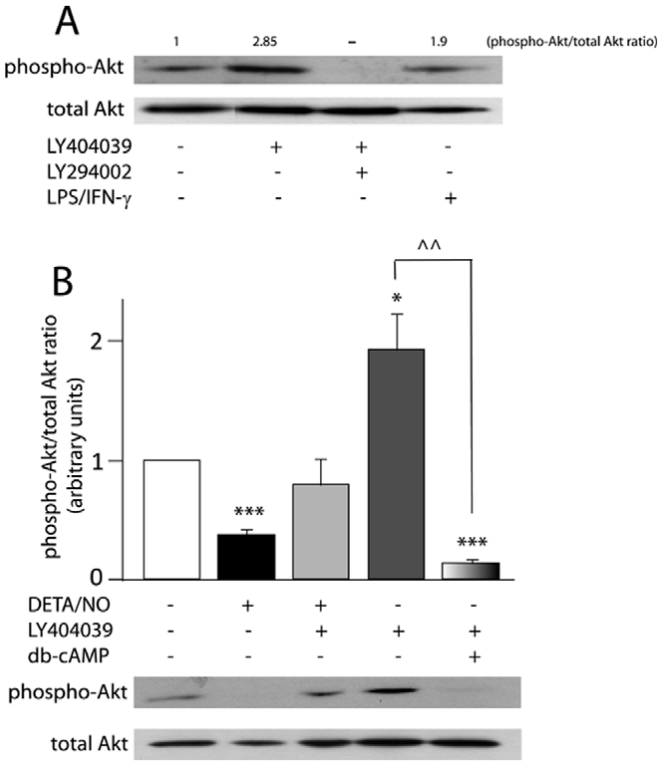
LY404039 avoids reduction in phospho-Akt levels caused by DETA/NO. Total cellular proteins were extracted as described in Materials and Methods from astrocytes exposed to 1 mM DETA/NO±100 µM LY404039±1 mM db-cAMP for 30 min, and 50 µg of protein extracts were assayed for phospho-Akt by western-blot. (A) Specificity of the western-blot was analyzed by incubating astrocytes with the PI3K/Akt pathway inhibitor, 20 µM LY294002, which completely prevented LY404039-induced Akt phosphorylation, and LPS/IFN-γ was used as positive control of Akt activation. Membranes were stripped and incubated with anti-total Akt antibody, which was considered loading control. (B) Data were expressed as phospho-Akt/total Akt ratio and related to control group. Bars represent the mean ± SEM of 4 independent experiments. *p<0.05, ***p<0.001 versus control, ∧∧p<0.01 versus LY404039.

To further support these results, we also assayed protein levels of phosphorylated-Akt (phospho-Akt) by western-blot. As a positive control of the technique, astrocytes were incubated with LPS/IFN-γ [29], which incremented the phospho-Akt/Akt ratio by 100% (Fig. 7A). LY404039 also induced Akt phosphorylation *per se* (Fig. 7A and B). As a negative control, astrocytes were co-incubated with LY404039+the PI3K/Akt pathway inhibitor, LY294002 (20 µM), which completely blocked Akt phosphorylation induced by the mGluR3 agonist (Fig. 7A).

We show that the NO donor significantly diminished the phospho-Akt/Akt ratio, whereas LY404039 prevented this effect (Fig. 7B), which concords with an anti-apoptotic role for phospho-Akt.

Interestingly, co-incubation with 1 mM db-cAMP blocked LY404039-induced Akt phosphorylation (Fig. 7B), suggesting that this second messenger exerts an inhibitory effect on the mGluR3- activated PI3K/Akt pathway.

## Discussion

Our present results show that three cell signaling mechanisms are involved in the anti-apoptotic actions of mGluR3 in NO-exposed rat astrocytes: i) reduction in cAMP levels as a consequence of adenylyl cyclase inhibition by mGluR3-coupled Gi proteins; ii) the PI3K/Akt pathway, which involves Akt phosphorylation and, in turn, a phosphorylating cascade that results in triggering of survival signals; iii) dimerization between p65 and c-Rel, transcription factors belonging to the NF-κB family, which can mediate the transcription of anti-apoptotic genes.

Activation of NF-κB proteins is a ubiquitous event in inflammatory processes that induces the expression of pleiotropic genes, from pro-inflammatory genes such as iNOS to anti-apoptotic genes such as Bcl-2. LPS/IFN-γ is known to induce NF-κB activity [29] leading to up-regulation of iNOS, tumor necrosis factor-α and cyclooxygenase-2 expression. Here, we found no effect of LY379268 on LPS/IFN-γ-induced p65 expression. Therefore, inhibition of LPS/IFN-γ-induced iNOS expression by LY379268 that we reported earlier [3] cannot be explained by any effect of the agonist on p65 nuclear levels. This suggests that other mechanisms must be involved in regulation of iNOS expression by mGluR3. Kozuka *et al.* (2007) observed NF-κB-independent iNOS induction [30] and Murphy *et al.* (1995) reported that mGluR activation inhibits cytokine-induced iNOS expression acting downstream of NF-κB translocation to the nucleus [31].

On the other hand, LPS/IFN-γ-induced IκBα inhibition was prevented by LY379268 and this agonist incremented IκBα expression *per se*. The evident uncoupling of p65 from IκBα results can be explained by the existence of other forms of IκB (IκBβ, IκBγ, IκBε) that are subjected to regulation different from that of IκBα [32]. These variants of IκB could avoid regulation exerted by LY379268, thus holding their diminished levels and allowing p65 to remain at the nucleus, even after cytosolic levels of IκBα have been restored. In fact, the association of IκBβ with sustained activity of NF-κB over time has been described, since the resynthesis rate of IκBβ is slower than that of IκBα [33]. Moreover, other authors also reported similar uncoupling of NF-κB from IκB activity [34].

As we mentioned above, an anti-apoptotic role for p65-c-Rel heterodimers has been proposed [24], [35]–[37], since their nuclear activity leads to transcription of Bcl-2 and Bcl-xL genes. Based on this background, we suggest that NO-inhibited and mGluR3 agonists-induced p65-c-Rel nuclear levels in astrocytes might support a protective role of these heterodimers and could be correlated with the modulation of Bcl-2 family proteins previously reported [3]. We also observed that the effect of mGluR3 agonists is specific for p65-c-Rel interaction, given that they did not modify free p65 nuclear levels. Since we found no synergic effect of LY379268 and LPS/IFN-γ on p65-c-Rel interaction, it is likely that both stimuli are converging at the same signaling pathway. This pathway could be PI3K/Akt, which is known to be induced by both agents [8], [38], tallying with our present data which show that both LPS/IFN-γ and the mGluR3 agonist activate Akt.

Here, we also demonstrated that both Akt activation and reduction in cAMP levels are required for mGluR3 cytoprotection. Although the cytotoxicity of cAMP is controversial, some studies support the hypothesis that a reduction in cAMP levels is a protective mechanism in the CNS. Faden *et al.* (1997) reported that group III mGluR agonists (which also inhibit adenylyl cyclase) reduce neuronal death induced by mechanical damage and that this effect is mediated by cAMP inhibition [39]. In fact, cAMP-induced PKA activity can promote apoptosis [40]. cAMP also limits membrane localization of phosphoinositide-dependent kinase-1 [41], a direct effector kinase of Akt. Concordant with these data, we show here that db-cAMP prevents mGluR3-induced Akt activation and blocks the protective action of LY379268 on astroglial viability.

Participation of Akt in the survival pathway triggered by mGluR3 was described earlier in astrocytes deprived of oxygen/glucose and its activity correlated with Bad inhibition, Bcl-xL induction [8] and TGF-β release [7]. Akt was shown to phosphorylate several proapoptotic proteins leading to suppression of death signals [42]. Our data indicate that NO reduces Akt activation, which may be one initial event in the triggering of the apoptotic cascade. Further, since PI3K/Akt activation precedes NOS up-regulation [38], [43], [44], NO might inhibit the PI3K/Akt pathway as part of a negative feedback mechanism. The restoration of DETA/NO-reduced phospho-Akt levels by LY404039 correlates with present data indicating that Akt activity is involved in cytoprotective actions of mGluR3 in astrocytes.

Despite the correlation between astrocyte death induced by both LPS/IFN-γ and NO, and the fact that NO has demonstrated to be the mediator of LPS/IFN-γ-induced cell death [3], signaling transduction mediators of LPS/IFN-γ and NO do not seem to be exactly the same ones. In fact, we have shown in the present work that, while LPS/IFN-γ induces Akt and NF-κB activation, NO inhibits both Akt phosphorylation and p65-c-Rel interaction. Although these inhibitory effects are coherent with the NO pro-apoptotic role, LPS/IFN-γ can also induce astrocyte death by activating a broad range of alternative pathways such as p38 MAPK and JNK [45]–[47], in spite of its effects on Akt and p65-c-Rel. Finally, LPS/IFN-γ stimulus is able to induce astrocyte death through NO production, although (possibly because of the side activation of Akt and p65-c-Rel) the quantity of dead astrocytes by LPS/IFN-γ is lower than that induced by NO [3]. This evidence indicates that mGluR3 activation would prevent LPS/IFN-γ-induced astrocyte death by reducing NO synthesis, whereas it could block NO-induced astrocyte death by keeping Akt and p65-c-Rel levels up.

**Figure 8:**
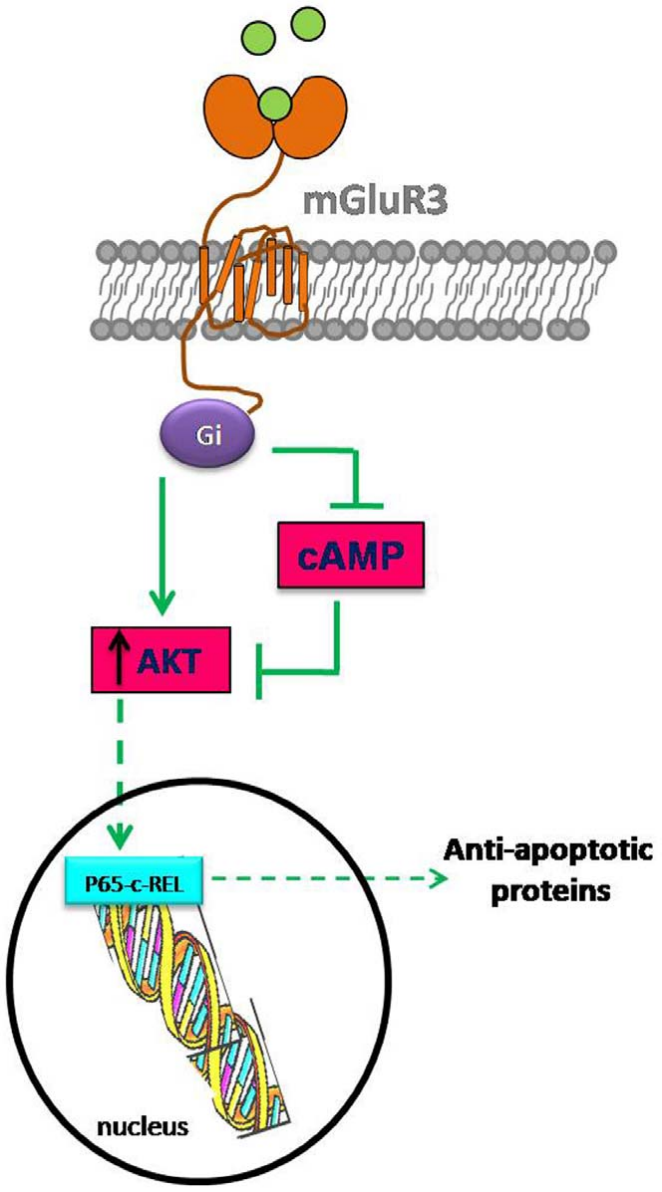
Proposed model for mGluR3 protective actions in astrocytes. mGluR3 activation by selective ligands couples to Gi protein activation, which can inhibit adenylyl cyclase activity and cAMP production and/or directly activate PI3K/Akt pathway. Alternatively, cAMP reduction could lead to Akt activation, because cAMP inhibits Akt phosphorylation. In turn, Akt might induce p65-c-Rel interaction at nucleus, which stimulates the transcription of anti-apoptotic genes such as Bcl-2, resulting in astrocyte protection from NO challenge.

In agreement with other authors [41], [48], our present results show that cAMP can inhibit Akt phosphorylation, and thus mGluR3-reduced cAMP levels could lead to Akt pathway activation. Since Akt was also reported to promote NF-κB activity [38], [44], [49], we propose that Akt activity might induce nuclear p65-c-Rel interaction, directing the synthesis of anti-apoptotic members of the Bcl-2 family and ultimately modulating the cell death cascade induced by NO in astrocytes (Fig. 8).

In summary, we identified novel aspects of the signal transduction system associated with mGluR3 activation in astrocytes, which may be responsible for the cytoprotective actions of mGluR3 agonists in NO-exposed astrocytes. Elucidation of the mechanisms activated by mGluR3 agonists may offer insights for potential future therapeutic strategies for the treatment of neurodegenerative disorders associated with high NO production and astrocyte loss.
